# A general mechanistic model enables predictions of the biological effectiveness of different qualities of radiation

**DOI:** 10.1038/s41598-017-10820-1

**Published:** 2017-09-07

**Authors:** Stephen J. McMahon, Aimee L. McNamara, Jan Schuemann, Harald Paganetti, Kevin M. Prise

**Affiliations:** 1 Centre for Cancer Research and Cell Biology, Queen’s University Belfast, Belfast, BT9 7AE Northern Ireland; 2 Department of Radiation Oncology, Massachusetts General Hospital, 30 Fruit St, Boston, MA 02114 USA

## Abstract

Predicting the responses of biological systems to ionising radiation is extremely challenging, particularly when comparing X-rays and heavy charged particles, due to the uncertainty in their Relative Biological Effectiveness (RBE). Here we assess the power of a novel mechanistic model of DNA damage repair to predict the sensitivity of cells to X-ray, proton or carbon ion exposures *in vitro* against over 800 published experiments. By specifying the phenotypic characteristics of cells, the model was able to effectively stratify X-ray radiosensitivity (*R* ^2^ = 0.74) without the use of any cell-specific fitting parameters. This model was extended to charged particle exposures by integrating Monte Carlo calculated dose distributions, and successfully fit to cellular proton radiosensitivity using a single dose-related parameter (*R* ^2^ = 0.66). Using these parameters, the model was also shown to be predictive of carbon ion RBE (*R* ^2^ = 0.77). This model can effectively predict cellular sensitivity to a range of radiations, and has the potential to support developments of personalised radiotherapy independent of radiation type.

## Introduction

Cancer radiotherapy has long been a highly personalised treatment, with significant research and technical development being devoted into better identifying, localising, and treating cancers with ionising radiation. In modern radiotherapy, patients are treated with individualised treatment plans administered by sophisticated delivery techniques such as Intensity Modulated RadioTherapy (IMRT) and guided by a range of advanced imaging modalities^1^. These advances in delivery have resulted in significant patient benefit by improving the targeting of radiation to the tumour while sparing surrounding normal tissue, enabling an escalation of treatment doses and corresponding improvements in clinical outcomes. Further technical advancement to refine these techniques remains a key area of research.

However, despite its high degree of geometric personalisation, radiotherapy has seen limited biological personalisation. For most cancers, the treatment dose and schedule are typically determined by the site (and potentially stage) of cancer alone, with the majority of patients receiving a ‘one size fits all’ treatment. While these schedules are often based on the results of very large clinical trials^2, 3^, their results typically assume a uniform radiation sensitivity across a whole population. This is known to be a significant oversimplification, as there is extensive evidence that cancers of the same type can have very different sensitivities to ionising radiation, a fact which is underscored by the complex genetic heterogeneity of cancer^4, 5^.

While it remains challenging to directly measure the sensitivity of clinical cancers in patients, there is extensive evidence that this variation is significant. Modelling of radiation response curves based on clinical data has suggested that the dose needed to control 50% of tumours (TD50) could have a standard deviation of 20–25%^6^, a range which is reflected in many *in vitro* studies of cellular radiation sensitivity^7–9^. Such a range means that any dose selected on the population level would certainly over- or under-treat large numbers of patients, impacting negatively on clinical outcomes. To address this, a better understanding of the biological drivers of clinical radiation response is required.

Numerous physiological factors have been linked to treatment outcome including clinical parameters such as tumour volume and stage, as well as micro-environmental factors such as the level of perfusion and resulting availability of nutrients and oxygen in the tumour. While there has been considerable interest in using a number of these factors to personalise treatment doses (particularly reduced oxygen, i.e. hypoxia^10^), few of these tools have made an impact on clinical practice. However, independent of the physiology of the tumour, cells have an intrinsic radiosensitivity, driven by their particular tissue of origin and any acquired mutations which impact on radiation response. Intrinsic radiosensitivity can be assayed *in vitro* through clonogenic assays, and has been shown to not only reflect the wide range of radiosensitivities which are observed clinically, but also to be a strong, independent predictor of an individual’s successful response to radiotherapy^7, 9, 11, 12^.

As direct measurement of intrinsic radiosensitivity remains challenging, techniques to predict it have the potential to significantly impact on treatment decisions. There is considerable interest in the identification of key mutations^13^ or gene expression signatures^14–16^ which identify differential radiosensitivities, but these approaches have shown limited success in generating translatable predictions. A particular challenge with these approaches is the very large data sets which are required to generate meaningful fits, as they typically do not take advantage of our underlying knowledge of radiation effects at a biological level, instead focusing on purely statistical approaches to identify trends.

The growing availability of advanced radiotherapy techniques which make use of heavy charged particles such as protons and carbon ions presents an additional challenge in this area. These heavy charged particles deposit their energy more densely (characterised by a high Linear Energy Transfer (LET)) as they pass through the cell, and are known to be more damaging than the X-rays conventionally used in radiotherapy for a given radiation dose (characterised by a Relative Biological Effectiveness (RBE)). A number of approaches exist to characterise the RBE of charged particles, including empirical modifications to the Linear-Quadratic dose response model^17–19^ as well as mechanistic models of radiation-induced cellular damage^20–23^. However, RBEs are known to depend on the underlying biology of the cells being irradiated, and as a result these models typically also require cell-specific information (such as dose-response information) to generate predictions. As uncertainties in how cells will respond to charged particle irradiation may significantly alter the expected clinical benefit of moving from X-ray to more expensive ion based therapies, better understanding of these effects could significantly impact on the allocation of these scarce resources.

Improved mechanistic models of fundamental cellular radiation responses offer an alternative approach to these problems. By integrating our knowledge of the underlying biology, models of radiation sensitivity can be generated which depend on known mechanistic determinants of radiosensitivity, such as DNA repair and cell cycle effects. In addition to constraining model parameters, these approaches can also leverage data from multiple endpoints simultaneously, rather than relying on only direct measurements of the endpoint of interest (e.g. cell survival). This approach may offer improved predictive power in novel systems without the need for abstract fitting parameters and direct survival measurements, provided the systems can be modelled with sufficiently low uncertainty.

As a first step towards the generation of cell-specific predictions of radiation sensitivity, we have recently published a mechanistic model that draws on measurements of fundamental processes such as DNA repair, chromosome aberration formation and mutation to predict cellular response. This model begins from initial distributions of DNA Double Strand Breaks (DSBs), and calculates the probability of different types of repair and misrepair, and the eventual fate of the cell. Significantly, this model does not make use of cell-specific fitting parameters, but rather defines parameters describing different processes common across a range of cell types (e.g. the rate of DNA repair by different pathways). Cell-specific predictions are then generated from these parameters based on specific phenotypic characteristics, such as whether the cells have functional Homologous Recombination (HR) and Nonhomologous End Joining (NHEJ) repair pathways. In previous work we have shown that this integrated model can characterise a range of biological endpoints, including DNA repair, chromosome aberration formation and survival in an initial dataset^24^.

**Figure 1:**
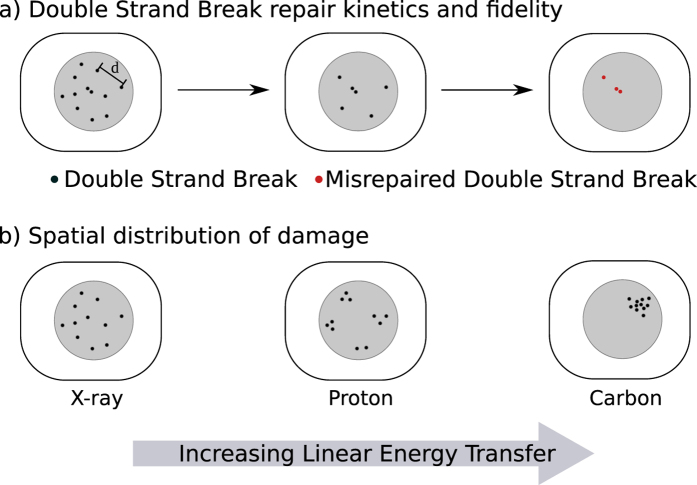
Schematic illustration of mechanistic DNA repair model, combining a biological model of DNA DSB repair (**a**) and a physical model of DSB distribution (**b**). The biological model begins from a distribution of DSBs, whose repair is simulated as a function of time. Each break can be repaired either correctly, or misrejoin with another nearby DSB with a rate which depends on their separation *d*, with a rate constant given as $\zeta (d)\propto {e}^{-\frac{{d}^{2}}{2{\sigma }^{2}}}$. The number and type of misrepaired DSBs can then be used to predict overall sensitivity. The physical model considers the distribution of tracks around charged particles, as illustrated in (**b**). While equal doses are considered to cause the same number of DSBs, these are uniformly distributed in the case of X-rays, clustered around a small number of tracks for protons, and extremely densely clustered around carbon ion tracks, as determined by their LET and radial dose distributions.

In this work, we assess the predictive power of the model by comparing its predictions of radiation sensitivity based on this mechanistic fit to a panel of over 800 radiation survival curves from the literature, encompassing X-ray, proton and carbon ion survival data. Predictions of X-ray sensitivity were made directly based on parameters established in the mechanistic fit without adjustment. Extension to charged particle therapies is achieved by linking the established biological model with a simple Monte Carlo simulation of energy distribution around charged particle tracks. Combining the two models involves the fitting of a single additional parameter to link physical energy deposition to DSB production, which was carried out by fitting to proton radiation response data. We then demonstrated that this model can be applied to carbon ion data without any direct fitting, highlighting how this mechanistic approach enables predictions of effects within a range of systems. The model is schematically illustrated in Fig. 1, and described in detail below.

## Results

### Model predictive power – X-rays

**Figure 2:**
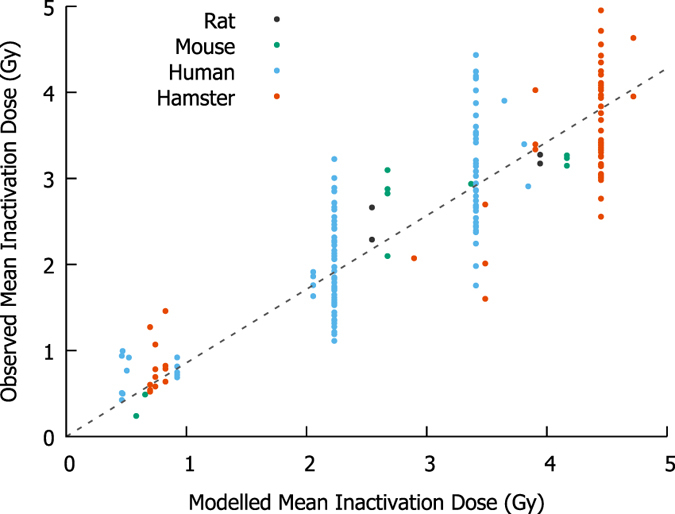
Comparison of modelled and observed cellular radiosensitivity to X-rays. Mean Inactivation Doses (MIDs) were calculated for a range of different cell types exposed to X-rays from several datasets^24–26^, using previously established mechanistic response parameters^24^. Without any cell-specific fitting parameters, good correlation is seen across the entire range of sensitivity, although considerable inter-experimental variation remains, even among notionally identical experimental conditions.

To test the predictive power of the model for a range of different cell lines exposed to X-rays, Mean Inactivation Doses (MIDs) were calculated for each of the unique X-ray experiments reported in two radiation response databases – Paganetti’s review of proton RBE^25^ and the Particle Irradiation Data Ensemble (PIDE)^26^, together with those from a previous study^24^. For each X-ray response curve in these datasets, the Mean Inactivation Dose (MID) was calculated from experimental observations, and compared to the sensitivity predicted by the established mechanistic model. The correlation between model predicted and observed sensitivity is shown in Fig. 2. Although this data represents a wide range of cell lines, with different species of origin, genetic alterations and exposure conditions, good correlation is seen across the whole range of radiosensitivities, with effective stratification according to both genetic defects as well as species and cell cycle phase during irradiation.

The overall correlation coefficient is *R* ^2^ = 0.74, indicating that the model accurately reflects the majority of variation in sensitivity between cell lines, without cell-specific adjustable parameters, suggesting that this model retains predictive power even in this larger, more heterogeneous dataset.

### Radial energy distribution and interaction

**Figure 3:**
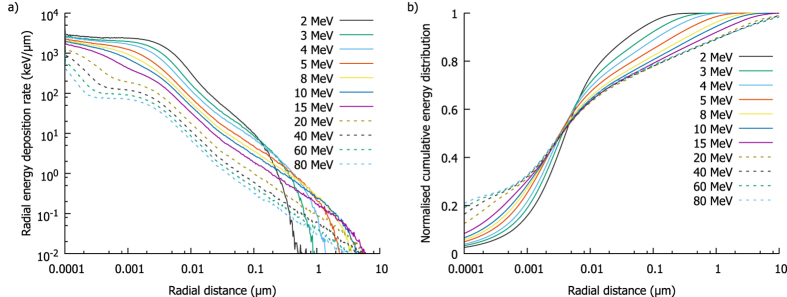
Radial energy deposition around protons of different energies. In (**a**), rate of energy deposition as a function of distance from the primary track shows an increasing amount of energy is deposited as particles slow, with the greatest density within a few nm of the track. However, (**b**) shows a normalised cumulative energy distribution as a function of distance from the track. Due to the small volume near the track the total energy deposited energy is relatively small, with a large fraction of deposited at long distances, even beyond 1 μm for the most energetic protons.

Calculation of model predictions for charged particle radiation requires calculation of the spatial distribution of energy around particle tracks, illustrated for protons in Fig. 3. Figure 3a shows the rate of energy deposition scored radially around the track, showing a clear maximum close to the charged particle and falling rapidly to roughly 10% at the edge of the track ‘core’ (~10 nm in radius). However, because of the extremely small size of the track core, the total amount of energy deposited at long ranges is not negligible. This can be seen in Fig. 3b, which plots the cumulative radial energy deposition. This shows that the track core represents less than 25% of the total energy deposition, with approximately half of the energy deposited more than 50 nm from the track, and a significant portion out to several μm for the most energetic protons.

Based on the assumption that DSB yield is directly proportional to energy deposit, these energy distributions can be converted to DSB distributions by dividing the energy in each bin by the energy required to create one DSB on average - *E*_*DSB*_. This parameter determines the density of DSBs within a track and thus the rate of intra-track interactions and the overall RBE.

**Figure 4:**
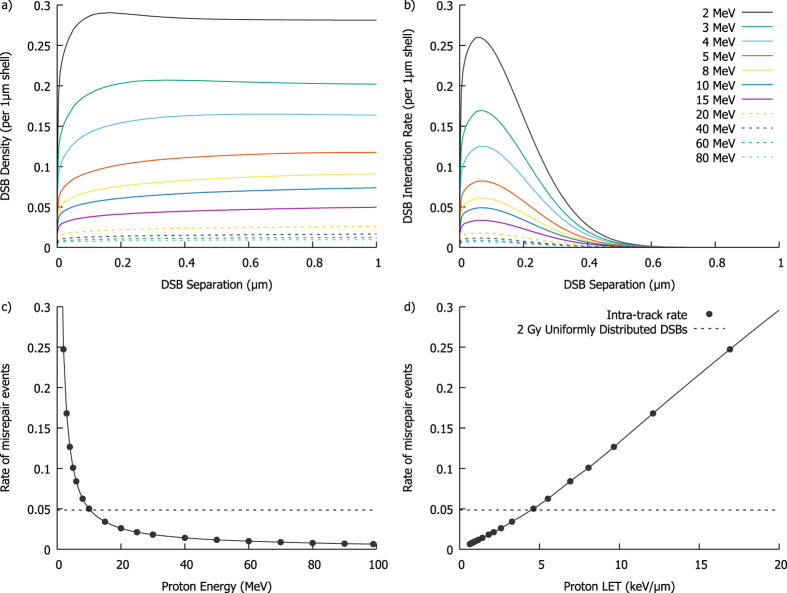
Double Strand Break (DSB) distributions and resulting misrepair rates. By converting the energy distributions from Fig. 3 into DSB distributions, the spatial distribution of damage around an ‘average’ DSB can be calculated. This is shown in (**a**), with a rapid rise of DSB count with range as greater portions of the particle track are encompassed. Panel (b) presents the resulting interaction rate, obtained by scaling the DSB density by ${e}^{-\frac{{r}^{2}}{2{\sigma }^{2}}}$, showing most intra-track interactions occur for DSBs separated by a few hundred nm. By integrating the interaction rates from (**b**), the total interaction rate can be obtained for different particle energies. The rate is plotted as a function of either proton energy (**c**) or LET (**d**). This rate increases steeply as the particle slows down, and is closely related to LET – although this relationship is slightly super-linear, due to the smaller total track radius at lower energies. The misrepair rate for 2 Gy of uniformly distributed DSBs is also plotted for comparison (dashed line), showing that even at moderate LETs (>5 keV/µm) intra-track effects can be a dominant contribution to misrejoining rates at clinical doses.

By integrating the distance between these DSBs both radially and along the track path, the expected number of DSBs separated by a given distance can be calculated. This distribution can then be divided by the number of DSBs to give the expected number of DSBs as a function of position around an ‘average’ DSB within the track. Figure 4a illustrates this for protons, using the *E*_*DSB*_ value obtained by fitting to proton RBE data as described below. These curves show a steep initial increase in DSB density with saturation at distances of a few hundred nm. More rapid build-up is seen for lower energy particles, due to their relatively dense track cores. The long-range saturation level is proportional to twice the LET of the particle, as large radial shells encompass the entire track both upstream and downstream of any break.

The total interaction rate of free ends created by DSBs is obtained by scaling these DSB densities by the interaction rate *ζ*(*r*) and illustrated in Fig. 4b. This shows that due to the relatively short range of DSB interactions misrepair events are dominated by DSBs which occur within ~200 nm of each other.

Integrating the rate distributions of Fig. 4b gives the total intra-track misrepair rate, *η*, which is plotted as a function of particle energy and LET in Fig. 4c and d, respectively. As lower energy particles have a higher LET and proportionately greater number of DSBs induced per unit track length, it can be seen that they have a much greater probability of intra-track misrepair events occurring. The relationship between interaction rate and LET can be seen to be slightly greater than linear, as at lower proton energies the radial distribution also becomes narrower, further reducing DSB separation, although this effect is relatively weak.

For comparison, Fig. 4 also plots the average misrepair rate for a uniform distribution of DSBs corresponding to an exposure of 2 Gy. This shows that while the intra-track misrepair rate is small for low LETs, at high LETs and low doses intra-track recombination events may be the dominant contribution to cell killing.

### Model predictive power – protons

By using these radial dose distributions to calculate intra-track misrepair rates, the model can be applied to charged particle predictions by the fitting of the single additional parameter *E*_*DSB*_ to link between the physical and biological models. In this work, this parameter was determined by fitting to the experimental proton RBE data reviewed by Paganetti^25^.

**Figure 5:**
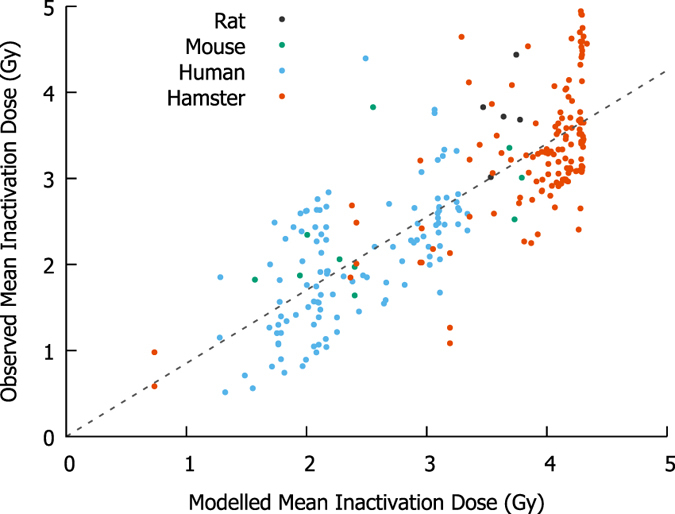
Comparison of modelled and observed cellular radiosensitivity to protons. By fitting a single parameter, *E*_*DSB*_ to scale from the radial energy data in Fig. 3 to DSB rates in Fig. 4, proton MID values can be calculated for the experiments reported in Paganetti^25^. Although the total level of noise increases, the overall correlation remains good (*R* ^2^ = 0.66), outperforming a simple linear scaling of sensitivity.

The best-fitting value was found to be 60.7 ± 14.0 keV, and this value was used to generate proton MID values for the different conditions which are compared to experimental observations in Fig. 5. While there is an increase in variation compared to the photon data, the overall correlation remains good (*R* ^2^ = 0.66), indicating that the model captures much of the underlying variation. This *E*_*DSB*_ is equivalent to a nuclear radius of *r*_*nuc*_ = 4.32 ± 0.2 µm for human cells, in line with typically reported values of 4 to 5 µm, supporting the biological rationale behind this model.

However, when predictions of the Mean Inactivation Dose RBE (*RBE*_*MID*_) are considered, the predictive power of the model appears to be limited (*R* ^2^ = 0.28), although this still significantly outperforms the approach of linearly scaling doses by a constant RBE which remain common in the clinic (p < 0.0001, t-test of correlation coefficient, t = 10.09, n = 258). However, robust validation of proton RBE predictions remains challenging due to the high degree of inter-experimental variability in dosimetry, experimental conditions and cell survival measurements. Because RBE values involve the ratio of two dosimetric parameters derived from these measurements, this uncertainty is often significantly greater than the magnitude of the effect. As a result, other purely empirical analytic models^17–19^ which directly use the observed cell α and β values as well as several empirical fitting parameters do not offer significantly better RBE predictions (*R* ^2^ values between 0.18 and 0.28, see supplementary information). This suggests that alternative approaches may be necessary to validate RBE models.

### Model predictive power – heavier ions

Because of the mechanistic nature of this model, it can be directly applied to heavier ions by using the appropriate radial energy distributions. Testing its predictions for heavier ions thus provides a valuable opportunity to test the model’s underlying assumptions and predictive power in a novel system. To test this, radial dose deposition curves were simulated for carbon ion tracks and used to calculate *D*_10_ values for all of the carbon ion exposures from the PIDE^26^. Illustrations of carbon ion DSB distributions and misrepair probabilities for comparison with Fig. 4 are presented in the Supplementary Information. The *E*_*DSB*_ value as obtained from the fit to the proton-only data was used in this analysis, to validate the model’s ability to extrapolate from effects in different systems.

**Figure 6:**
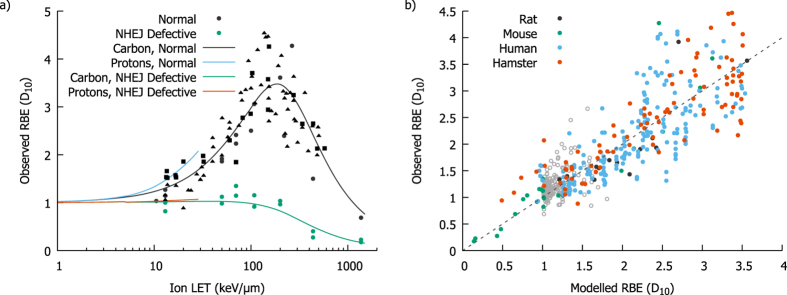
Model predicted Relative Biological Effectiveness for protons and carbon ions. The LET-dependence of RBE for carbon ions is shown in (**a**) for both repair competent and incompetent Chinese hamster cell lines (CHO: squares, V79: triangles, others: circles), compared to experimental observations. Good agreement is seen, correctly predicting the turnover at high LET and the relative lack of sensitisation seen in NHEJ-defective cells, despite the model only using the proton data to determine *E*_*DSB*_. Proton RBE is also shown, for comparison. An overall correlation of the modelled and predicted RBE is shown in (**b**). While the predicted variation in RBE for protons is swamped by the uncertainty (grey points) good correlation is seen across the full range of RBE values for carbon (coloured points).

In Fig. 6a, the LET-dependence of carbon *D*_10_ RBEs are shown for the frequently-used Chinese hamster cell lines CHO and V79, as well as a NHEJ-defective derivative line. A range of characteristics emerge from this model which would not be predicted by simply extrapolating empirical proton RBE models. These include the slightly lower RBE at a given LET for carbon ions than protons, the turn-over in carbon RBE at high LETs, and the lack of RBE in NHEJ-defective cells. The LET dependence of proton RBE is also shown, showing that the model also predicts the higher effectiveness of lighter ions at the same LET.

Figure 6b presents a correlation plot of model predicted and experimentally observed $RB{E}_{{D}_{10}}$ for both protons and carbon ions. While the proton data are dominated by experimental uncertainties and show limited correlation, the broader range of values for the carbon ion data provides a clearer test of the model with reduced sensitivity to experimental uncertainties. As with the MID predictions above, good correlation is seen across the whole range of observed RBEs, with a best-fitting slope coefficient of 1.00 ± 0.01 and a correlation coefficient of *R* ^2^ = 0.77. This suggests the model accurately captures a large degree of the underlying variation, particularly in light of the fact that no fitting was carried out either to the baseline X-ray data for either population or any of the carbon ion exposure data, demonstrating the ability of this model to extrapolate between different exposure conditions.

### Linear-Quadratic Model Parameters

**Figure 7:**
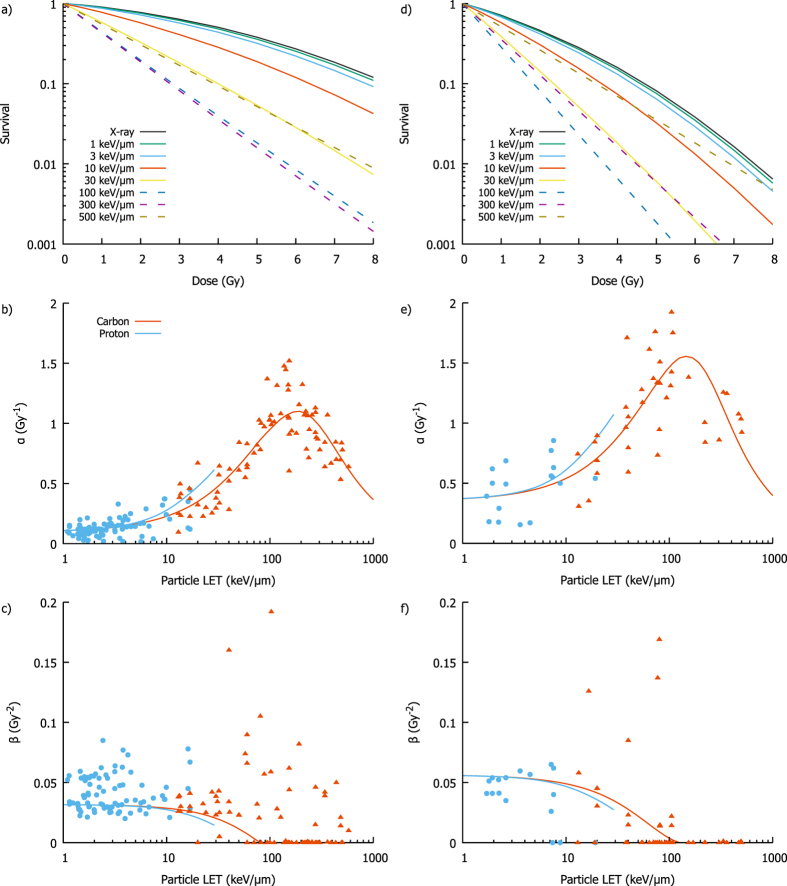
Illustration of model predicted LQ parameters for proton and carbon ions. Model predicted survival curves for asynchronous hamster cells with low $\frac{\alpha }{\beta }$ ratio are shown in (**a**), for exposures to a range of LETs, including X-rays, protons (solid lines, LET < 30 keV) and carbon ions (dashed lines, LET > 30 keV). As is seen experimentally, survival curves become steeper with increasing LET, eventually becoming linear at very high LET. Model predicted LQ parameters are presented in (**b** and **c**). *α* can be seen to increase to a maximum and then fall, mimicking the trend in overall RBE, while *β* is relatively constant at low LET before falling as track numbers are reduced. Similar trends are presented in (**d**–**f**) for normal human cells, with a relatively high $\frac{\alpha }{\beta }$ ratio, showing that the trends are qualitatively similar across cells of different radiosensitivities.

In addition to overall sensitivity parameters, the model is capable of generating full survival curves and corresponding LQ model parameters for different cell lines and exposure conditions. Examples of this are presented in Fig. 7, showing modelled radiation survival curves for radio-resistant Chinese hamster cell lines $(\frac{\alpha }{\beta }\approx 4Gy)$ and relatively radio-sensitive normal human cell lines $(\frac{\alpha }{\beta }\approx 10Gy)$ for a range of LETs delivered by X-rays, protons and carbon ions. In agreement with experimental observations, it can be seen that as particle LET increases, the curves become progressively steeper, losing the ‘shoulder’ characteristic of linear-quadratic dose responses, until at high LETs they become linear. These remain linear at LETs above the maximum effectiveness, but with progressively shallower slopes, as the probability of cells being exposed to even a single track becomes the major factor dominating survival.

LQ model parameters were extracted from these curves and compared to experimental observations. The *α* parameter closely follows the dependence of RBE as a whole, reflecting the increasing steepness of these curves, and is in broad agreement with observed values despite significant noise, particularly at low LETs. By contrast, *β* is initially relatively flat before falling to zero at high LETs where curves are purely linear. Comparison with experimentally observed *β* values is difficult as the dose ranges used in high LET exposures are typically limited, giving a high degree of covariance between *α* and *β* and corresponding increases in uncertainty. Thus, while over half of the dose-response curves at LETs above 50 keV/μm has zero *β* components, the remainder show significant variation which may be dominated by experimental uncertainties. However, the model successfully recapitulates overall trends in both the magnitude and shape of the dose-response curve with increasing LET.

## Discussion

The lack of predictive models of individual radiosensitivity remains a significant outstanding problem in radiotherapy, both in terms of the overall sensitivity of tumours as well as the relative benefit which could be expected from moving to new approaches such as charged particle therapies.

While there is considerable interest in the identification of key mutations or broader gene expression signatures which drive sensitivity^27, 28^, both of these approaches face the challenge of generalisability and do not take advantage of our extensive knowledge of the underpinnings of radiation response. By contrast, in this mechanistic model, the radiation sensitivity of a broad range of cell types in a range of experimental conditions is predicted based on a compact set of 10 parameters, which have been fit to a range of functional experimental endpoints (Table 1). These parameters show good predictive power, across a broad range of radiation sensitivities.

**Table 1:** List of parameters used in this work, and best fit parameter values from models.

Parameter	Meaning	Value
**Charged particle model parameters – Fit in this work**		
*E*_*DSB*_	Average energy required to produce 1 DSB	60.7 ± 14 keV
**Cellular parameters – Fixed from mechanistic model** ^24^		
**DNA Repair Parameters**		
	DNA Damage Yield	5.738 DSB/Gy/Gbp
***λ***_***F***_	Fast Repair Coefficient	3.6 ± 0.6 hours^−1^
***λ***_***S***_	Slow Repair Coefficient	0.15 ± 0.02 hours^−1^
***λ***_***M***_	MMEJ Repair Coefficient	0.0084 ± 0.0015 hours^−1^
***p***_***c***_	Complex break probability	0.42 ± 0.03
***p***_***f***_	Repair Failure Probability	0.67 ± 0.09
***σ***	Misrejoin range	0.0428 ± 0.0005 R_nuc_
***μ***_***NHEJ***_	NHEJ Fidelity	0.985 ± 0.002
***μ***_***MMEJ***_	MMEJ Fidelity	0.465 ± 0.05
**Survival Parameters**		
***ψ***	Mitosis Sensitivity	0.014 ± 0.002 break^−1^
***φ***	Apoptosis Sensitivity	0.0085 ± 0.001 break^−1^

All of the DNA repair and survival model parameters are taken from a previous publication without additional fitting^24^, while *E*_*DSB*_ is obtained from a fit to proton data in this work.

A major challenge in further model refinement is the degree of uncertainty in experimental radiobiological data, even for notionally identical exposures, which is a major factor in the reduction in X-ray correlation coefficient compared to previous work^24^. For example, in the large group of hamster cells with identical predicted sensitivity in Fig. 2, there are 28 different experimental reports of exposures of asynchronous V79 hamster cells with X-rays, which span a broad range of sensitivities (MIDs ranging from 2.8 to 4.9 Gy, with a coefficient of variation of 16%). Similar variability is seen in other cell lines, suggesting that these experimental uncertainties may represent 60% or more of the unexplained variation in sensitivity. These experimental uncertainties include differences in cell handling which are not typically reported in detail (e.g. cell cycle differences resulting from different times from plating to irradiation^29^), systematic and random experimental uncertainties (a recent report indicated laboratory radiation sources often have calibration errors of 10% or more^30^) and differences in irradiation protocol which are not currently implemented including dose rate and any RBE effects seen with low energy X-rays^31^).

In light of this uncertainty, more stringent model testing can be achieved by applying it in other areas. In this work, we considered the extension to exposures by charged particles, specifically the data on proton RBE collected by Paganetti^25^ and carbon ion exposures from the PIDE database^26^. To achieve this, we modelled the distribution of DSBs around particle tracks, fitting only a single additional parameter, *E*_*DSB*_, to link energy depositions calculated in the physical Monte Carlo to the biological model. The performance of this fit was comparable to many other models of proton RBE^17–19^, despite only involving a single charged-particle related fitting parameter and none of the cell-specific values required by other models. Moreover, good correlation was obtained for predictions of sensitivity to both protons and carbon ions, despite only fitting to the proton data, with the model accurately predicting the difference in effect between protons and carbon ions, and the turnover of RBE at high LETs. The degree of experimental uncertainty remains significant, particularly in proton RBE measurements, but overall the correlations were good (MID predictions have a correlation coefficient of *R* ^2^ = 0.8 across all data).

This correlation may potentially be further improved through the incorporation of a more granular phenotypic model, allowing for mutations which partially disrupt processes such as DNA repair, rather than the current binary model which is applied. In addition, while the overall performance of this model is high in *in vitro* data, several aspects remain to be improved before it can feasibly be implemented in a clinical setting. For *in vitro* data in cell lines such as the ones reported in the included studies, the phenotype can be established through experimental measurements which are not feasible in a clinical setting. As a result, alternative methods may be needed to generate these phenotypes in a predictive way, which may include the use of small targeted signatures where known genes in, for example, DNA repair pathways could be linked to specific phenotypic parameters. This would enable the model to be linked to clinical data from, for example, biopsies. In addition, while intrinsic radiosensitivity is a predictor of overall response^9, 12^, it would be desirable to expand the model to consider the responses of cells in a multicellular 3D system, enabling a consideration of factors such as changes in cell cycle distribution and radiosensitivity which occur upon the transition from *in vitro* to *in vivo* scenarios.

This approach may also benefit from more realistic models of DSB formation and resulting repair kinetics. At present, DSB yields depend only on energy deposition and consider discrete classes of ‘simple’ and ‘complex’ damage. In reality, it is known that a broad range of DNA damage types are possible, reflecting both the nature of break and where it occurs within the nucleus. This is known to be particularly sensitive to the type of radiation used, with a trend towards much more complex DSBs being seen in high LET irradiation which is believed to contribute to RBE^32^. In addition, the model currently uses the same simple exponential kinetics for repair for X-rays and all ions. This is known to be a simplification, as when misrepair events occur, there can be a significant delay in the repair of unpaired free ends, leading to more persistent damage and slower repair kinetics. These effects have been neglected here as within the current model formulation they do not impact on late outcomes such as survival for single-fraction acute exposures as studied here, but if there are other temporally-dependent processes which are not incorporated in this model these effects may become significant.

The inclusion of these assumptions may make it somewhat surprising that the current model performs well for heavy ions while treating all DSBs and resulting misrepaired lesions based on the X-ray response alone. However, there are some suggestions that these effects may be significant for some cells – such as homologous recombination defective cells, where RBEs are systematically under-estimated by the model – which suggests it would be desirable to link the model to more realistic DSB damage distributions provided by appropriate biophysical models, supported by more accurate models of DNA repair which take into account the effects of DSB interactions and misrepair on the repair kinetics and other mechanistic endpoints.

Despite these limitations, it can be seen that this mechanistic model of radiation response has good predictive power, having been validated against a set of over 800 experimental measurements of radiation sensitivity and showing good predictive power across a broad range of cell lines and conditions. This model approach may provide a valuable foundation for future investigations to understand radiation effects on tissue, as well as tailor individual treatments by incorporating more realistic models of individualised radiation responses.

## Methods

This work validates and extends a previously developed mechanistic model of DNA repair following ionising radiation exposures, by testing its predictive power in two new large datasets containing both X-ray and charged particle exposures. The model is schematically illustrated in Fig. 1, and key aspects are summarised below. Further details can be found in a previous publication^24^, and a full implementation of the model as used for the analysis presented in this work is available in the Supplementary Information.

### Model overview – DNA repair

This model begins from DNA double strand breaks (DSBs) as the initiating event in radiation-induced cell killing. Following exposure to ionising radiation, each cell contains a number of DSBs, *N*_0_, which is proportional to the dose delivered to the cell and the number of DNA base pairs within the cell (which varies with species and cell cycle stage as DNA is replicated). Each DSB is randomly assigned to be either ‘simple’ or ‘complex’, with some probability *p*_*c*_ at the time of creation. These DSBs can be repaired by one of three processes – Nonhomologous End Joining (NHEJ), Homologous Recombination (HR) and Mismatch End Joining (MMEJ), depending on the cell cycle phase and genetic background. In a repair-competent cell, simple DSBs are repaired by NHEJ throughout the cell cycle, while complex DSBs are repaired by NHEJ in G1, and HR in later phases when DNA has been replicated. In cells with repair defects, a proportion of DSBs which attempt to repair by a defective pathway will fail with probability *p*_*fail*_, and instead have to be repaired by the backup MMEJ pathway.

Each repair process repairs DSBs with exponential kinetics giving the total number of DSBs as $N(t)={N}_{0}({p}_{f}{e}^{-{\lambda }_{f}t}+{p}_{s}{e}^{-{\lambda }_{s}t}+{p}_{m}{e}^{-{\lambda }_{m}t}\,)$ where *p*_*x*_ and *λ*_*x*_are the probability and rate associated with each repair pathway. In addition to determining the kinetics of repair, each of these pathways has an associated fidelity, *μ*_*x*_, which indicates the probability with which the pathway will correctly repair a given DSB. HR has the highest fidelity, taken as 1, while MMEJ has the lowest fidelity, of 0.445. For each DSB that is repaired, this probability is randomly sampled, giving an associated rate of misrepair.

In addition to misrepair resulting from incorrect processing of a single DSB, binary misrepair can occur where free DNA strand ends from different DSBs are joined. Such events can be very significant for determining a cell’s fate following radiotherapy, as they can lead to significant genetic loss or the formation of chromosome aberrations. The process of misrepair is modelled in a spatially dependent fashion – as illustrated in Fig. 1, free DNA ends interact with a rate of $\zeta (d)\propto {e}^{-\frac{{d}^{2}}{2{\sigma }^{2}}}$, where *d* is the separation between the two ends and *σ* is a characteristic rejoining range. This gives a maximum rate for the two free ends created by a single DSB, which are initially adjacent, with a falling range as the separation between free ends increases.

For any given DSB, it can be shown that the probability of its two free ends rejoining correctly is given by ${P}_{correct}=\frac{1-{e}^{-\eta }}{\eta }$, where $\eta =\sum _{i=1}^{{N}_{0}-1}2{\zeta }_{i}$ is the sum of the interaction rates between a free end in a given DSB with the remaining *N*_0_ − 1 DSBs. For a uniform distribution of DSBs within a spherical nucleus, the average value of *η* for two randomly placed DSBs has an analytic form which depends only on the number of DSBs, the radius of the nucleus and the rejoining range *σ*, denoted as *θ*(*R*, *σ*)^24^. For other non-uniform distributions, *η* can be numerically calculated. The final probability of correct repair for a given DSB can then be given as ${P}_{correct}={\mu }_{x}(\frac{1-{e}^{-\eta }}{\eta })$, where *μ*_*x*_ is the process-specific repair rate. For a given exposure, this can be used to calculate the total number of misrepaired DSBs, which compares well to experimental observations^24^.

In addition to predicting the raw number of misrepaired events, the spatial component of the model can also be applied to predict the types of genetic aberrations resulting from these events. The first relevant classification is whether the event is *intra-* or *inter-* chromosomal: that is, are the two rejoining DSBs on the same or different chromosomes. As a simplified model, chromosomes are modelled as spherical sub-regions of the nucleus with radius ${r}_{c}=\frac{R}{\sqrt[3]{{n}_{c}}}$, where *n*_*c*_ is the number of chromosomes. While this neglects variations in chromosome size and packing that may impact on the rates in any given chromosome, it represents a useful average across a whole nucleus. The probability of intra-chromosomal events is then given as the ratio of the rate of interaction with another DSB within the chromosome volume to that across the nucleus as a whole: ${P}_{intra}=\frac{\theta ({r}_{c},\sigma )}{\theta (R,\sigma )}$.

These aberrations can also be classified as symmetric or asymmetric. This is defined based on whether the rejoined chromosomes are correctly aligned around their centromeres, with symmetric rejoining leaving two centromere-containing chromosomes while asymmetric rejoining leaves chromosomes with multiple or no centromeres, which are known to be incompatible with cell survival. As the free ends which correspond to each type of repair are otherwise identical, this is modelled simply as a random choice between each type of alignment, *P*_*asym*_ = 0.5.

Asymmetric intra-chromosome events lead to the deletion of genetic material. The size of this deletion is known to be very significant in determining cell viability. The rate of deletions less than a given size can be calculated in a similar fashion to the rate of inter-chromosome aberrations. If it is assumed that the genetic separation between two breaks increases monotonically with physical separation, then the size of a deletion in base pairs is given by $D=\frac{2L{r}_{D}^{3}}{{R}^{3}}$, where L is the total length of all chromosomes (in base pairs) and *r*_*D*_ is the separation between the involved DSBs. The rate of deletions smaller than *D* is then given by the fraction of DSBs within a chromosome which occur at a distance of *r*_*D*_ or less, which is given by ${P}_{del < D}=\frac{\theta ({r}_{c},\sigma ,{r}_{D})}{\theta ({r}_{c},\sigma )}$, were *θ*(*r*_*c*_, *σ*, *r*_*D*_) is a generalisation of the average interaction rate of two randomly placed DSBs within a sphere of radius *r*_*c*_ to only consider DSBs separated by less than a distance *r*_*D*_, the analytic derivation of which has been previously presented^24^.

Finally, in the G2 phase of the cell cycle when DNA has been replicated, there is also the possibility to observe inter-arm effects, where rejoining occurs across the centromere. While these events are hard to identify in G1, in G2 they are highly visible due to the deformation of the sister chromatid. For a DSB at a distance *b* (in base pairs) from the centromere, the probability of an intra-chromosome event also being inter-arm can be calculated as ${P}_{interArm}(b)={P}_{del > b}$, using the formulation above. Summing over all possible DSBs within a chromosome then gives a rate of ${P}_{interArm}=\frac{{l}_{c}}{L}{\int }_{0}^{0.5{l}_{c}}{P}_{del > b}db$.

Taken together, when repair is completed, the number of misrepaired DSBs, dicentric chromosomes (given by asymmetric intra-chromosome events), large deletions and inter-arm events (G2 only) are given by:

1 $${N}_{mis}={N}_{0}(1-{P}_{correct})$$

2 $${N}_{dic}=0.5\,{N}_{mis}(1-{P}_{intra})$$

3 $${N}_{del}=0.5{N}_{mis}{P}_{intra}(1-{P}_{del < D})$$

4 $${N}_{interArm}={N}_{mis}{P}_{intra}{P}_{interArm}$$

where D is set to 3 Mbp, based on established measurements of chromosome aberrations^33^. These model predictions have been fit and validated against a range of experimental observations, demonstrating broad applicability^24^. Predictions have also been produced for rates of mutation, but are not included here for brevity.

### Model overview – survival

Chromosome aberrations are one of the key drivers of cell death, particularly dicentrics, large deletions causing loss of significant genetic material, and inter-arm events in G2 preventing chromosome separation. Taking the predicted rates of dicentrics and deletions as average rates and assuming these events are Poisson distributed, we can then model survival in non-cycling cells as $S={e}^{-{N}_{dic}-{N}_{del > 3MBP}}$ for G1 and $S={e}^{-{N}_{dic}-{N}_{interArm}}$ for G2 (neglecting the probability of multiple large deletions in G2 rendering both daughter cells non-viable, which is a relatively rare event at clinically-relevant doses).

In cycling cells, two further forms of death are modelled – G1 arrest and apoptosis, and mitotic catastrophe. For simplicity, both processes are modelled as simple exponential functions of the number of DSBs present during G1 or M phase respectively, based on experimental observations. Mitosis is modelled for cells irradiated in G2 or M phase as ${S}_{mitosis}={e}^{-\varphi {N}_{m}}$, where *N*_*m*_ is the initial number of DSBs in M phase and *ϕ* is a rate parameter common across all cells. Similarly, apoptosis is measured as ${S}_{apoptosis}={e}^{-\psi {N}_{G1}}$, where *ψ* is a common parameter and *N*_*G*1_ is the number of DSBs present when the cell begins cycling in G1. While mitotic catastrophe is a common feature of all cell lines, G1 arrest and apoptosis is an active process, depending on p53 and other genes being functional. To reflect this, individual cell lines have been identified as G1 arresting or not, with cells which do not arrest in G1 not being subject to the apoptotic death rate.

The above model has been implemented in previous work and fit to and validated against a range of experimental measurements^24^, giving a set of model parameters outlined in Table 1. These parameters were used for all of the predictions generated in this work, without any additional fitting.

### Particle RBE characterisation

While the above approach has been validated for X-rays, it can be readily extended to DSB distributions caused by charged particles. As illustrated in Fig. 1, rather than considering a uniform distribution of DSBs by X-rays, DSBs now cluster around individual track paths. This leads to denser DSB clustering within the track path, which increases the value of *η* compared to a uniform exposure, and in turn increases the rate of misrepair.

To calculate *η* for a given track structure, the distribution of DSBs created by the track must be determined, which can in turn be used to calculate the average distribution of separations between DSBs. Once this distribution has been obtained, *η*_*track*_ can be calculated for the intra-track DSB interactions, and this can then be added to the *η* value associated with all DSBs caused by other independent tracks throughout the nucleus to give a total misrepair rate.

Radial track structures were modelled using Geant4 10.2 (patch 2)^34^, and the Geant4-DNA toolkit to provide precision at low energies^35, 36^. Protons and carbon ions at energies ranging from 1 to 100 MeV and 2 to 200 MeV/A, respectively, were directed along the central axis of a cylindrical water phantom, with radius 200 μm and depth 22 μm consisting of a central 2 μm scoring region and 10 μm build-up regions on either side to ensure adequate scattering. These volumes were selected following preliminary simulations with high-energy primary particles to estimate track diameters, and a small central scoring region was chosen to minimise the impact of primary particle scattering and maintain radial symmetry. Within the scoring region, energy deposition from both the primary particle and secondary electrons was recorded and scored in cylindrical bins according to distance to the primary proton track (recorded as it entered the scoring region), giving a radial energy distribution. The energy distribution was scored in logarithmic bins, with the smallest bin radius of 0.1 nm, and 100 bins for each factor of 10 change in radius (equivalent to a 2.33% increase in width in each successive bin). Primary counts ranged from 2000 to 20000 for protons and 600 to 20000 for carbon ions, depending on particle energy.

The model assumes that all radiation types create the same total number of DSBs per cell per unit dose: 5.738 /Gy /GBP, as determined in previous studies^24^. While these are distributed uniformly in the case of X-rays, ion radial energy distributions were converted to DSB distributions by assuming that the probability of a DSB being induced was directly proportional to the amount of energy deposited in a volume. That is, for a given volume the number of DSBs is given by $\frac{E}{{E}_{DSB}}$, where *E* is the energy deposited in a volume and *E*_*DSB*_ is the energy which leads to a single DSB on average. It should be noted that *E*_*DSB*_ is independent of the target volume under consideration – instead, it is assumed that this much energy causes 1 DSB whether it is deposited uniformly across the whole nucleus, or locally within some small sub-volume.

*E*_*DSB*_ cannot be determined from the DSB yield per cell alone, as this does not specify the target geometry. Instead, *E*_*DSB*_ is treated as a model parameter, and fit as described below. Because the current model sets a relationship between the dose to a nucleus and the number of DSBs, fitting *E*_*DSB*_ also determines a value for the volume of the nucleus. That is, ${N}_{D}=\frac{D\,{V}_{nuc}}{{E}_{DSB}}$, where *N*_*D*_ is the number of DSBs induced by a dose *D*, and *V*_*nuc*_ is the volume of the nucleus. For human cells, where a dose of 1 Gy induces 35 DSBs^37^, the volume in μm^3^ is given by ${V}_{nuc}=5.61{E}_{DSB}$, where *E*_*DSB*_ is measured in keV. This gives a nuclear radius, measured in μm, of ${r}_{nuc}=1.1\sqrt[3]{{E}_{DSB}}$, which provides a useful bound on the credibility of the *E*_*DSB*_ parameter.

Once radial DSB distributions are generated, the average separation between DSBs generated by a typical track can be calculated. Assuming radial symmetry in the energy distribution, each radial bin is subdivided into a series of small angular segments with a constant DSB density. A 2D distribution of DSB separations is then obtained by calculating the normalised sum of DSBs present in segments at different distances. Finally, the full 3D separation is obtained by assuming the DSB density is constant along the track length, and scaling the density of DSBs at each distance as appropriate. An implementation of this code is presented in the supplementary information.

The average interaction rate of DSBs within a track can then be calculated by summing *ζ* at each radial position, weighted by the expected number of DSBs with that separation. This sum then provides a *η*_*track*_ value, defining the intra-track DSB interaction rate – that is, the rate at which two DSBs from a single track will misrejoin. As it only considers DSBs from within a single track it is characteristic of the particle type and energy, but independent of the total number of tracks (and thus dose) delivered to the cell. This rate can be combined with the inter-track interaction rate, given by the whole-nucleus *η* rate defined above and the total number of DSBs present to give a total correct repair probability of ${P}_{correct}=\frac{1-{e}^{-\eta -{\eta }_{track}}}{\eta +{\eta }_{track}}$. This can be used to make cell- and particle-specific survival predictions to enable the calculation of RBE.

For simplicity, in this work the value of the parameter *η* associated with interactions of DSBs from different tracks across the whole nucleus is calculated assuming uniformly distributed DSBs. This is equivalent to the assumption that there is no correlation between the positions of DSBs caused by independent tracks. While this assumption correctly predicts the average separation of DSBs for all radiations, at very high LETs it under-estimates the variance in DSB clustering and thus in cell survival. However, as all experimental studies considered here focus on mean survival, this does not impact on the model fit. In addition, only the impact of intra-track events on the total rate of misrepair is modelled, with the probability of misrepair events leading to different classes of chromosome aberrations and various sizes of deletions still being calculated from the established biological model.

Finally, at very high LETs each track will potentially cause multiple DSBs and low dose exposures may only consist of a few particle traversals, giving rise to over-dispersed, non-Poisson initial damage yields. To account for this, when the expected number of DSBs per track traversing the nucleus is greater than 0.5 (based on LET and *E*_*DSB*_), cellular responses to discrete numbers of particle traversals are calculated. An average response is then returned, weighted assuming a Poisson distribution of tracks around the mean number which delivers the prescribed dose.

### Data selection and analysis

Two sources of cell survival data were used to test the model’s predictive power and extend its predictions to charged particle therapies. The first is the proton RBE dataset published by Paganetti^25^, which presented a comprehensive review of published proton RBE studies, with both X-ray and proton α and β values for linear-quadratic survival curves, and corresponding experimental conditions. The second source was the Particle Irradiation Data Ensemble (PIDE), produced by GSI^26^, which contains similar data for a range of ion exposures, from which the carbon ion exposure data was extracted.

Both datasets were filtered to extract experiments investigating single-fraction *in vitro* exposures of adherent cells in oxic conditions. Additional filters were applied to exclude studies where charged particles had ranges less than one cell diameter, or where the dose range was extremely limited (all data points < 2 Gy). The resulting dataset contained a total of 202 X-ray experiments, 260 proton experiments, and 325 carbon ion experiments. For each experiment, the following data was extracted: the cell line used, the particle used, the LET (if appropriate), the cell cycle phase at the time of irradiation (including the delay until cells were replated/released from cell cycle arrest, if relevant), and the fitted α and β response parameters. Cells irradiated in asynchronous conditions were treated as having a 2:1 distribution between G1 and G2, as full cell cycle information was typically not available. This approximation is believed to be valid as, in most cell lines, radiosensitivity varies smoothly through S phase from the G1 sensitivity to the G2 sensitivity, allowing asynchronous populations to be reasonably approximated by a mixture of G1 and G2 cells^38^.

The cell line used in each experiment was characterised according to the following parameters – its genome size, its chromosome count, the presence of functional HR and NHEJ, and the activity of its G1 damage arrest. For HR, NHRJ and G1 arrest, these were treated as simple binary variables, where processes are either fully functional or fully defective. The variables of interest and their format are presented in Table 2, and a full listing of all experimental conditions modelled is included in the Supplementary Information.

**Table 2:** List of cell-specific characteristics which are used to characterise cells in this model.

Parameter	Description
Genome Size	Length of haploid genome in GBP
Chromosome Number	Total number of chromosomes in cell
NHEJ Repair Capacity	Availability of NHEJ repair pathway
HR Repair Capacity	Availability of HR pathway
G1 Arrest Function	Availability of G1/S phase damage arrest checkpoint
Cell Cycle Phase	Cell cycle phase (Single specified phase or asynchronous)

All parameters are obtained from the literature and used to predict cellular radiosensitivity without free fitting parameters.

For each experimental dataset, we calculated the Mean Inactivation Dose (MID) and *D*_10_ (the dose at which 10% of cells survive, typically used to characterise high-LET radiation sensitivity) to provide single dosimetric metrics to characterise the radiation responses of the cells. The MID is the dose required to kill an ‘average’ cell in the population, and can be defined as the area under the dose response curve, given by:

5 $$MID={\int }_{0}^{\infty }{e}^{-\alpha D-\beta {D}^{2}}dD=\frac{{e}^{\frac{{\alpha }^{2}}{4\beta }}\sqrt{\pi }Erfc(\frac{\alpha }{2\sqrt{\beta }})}{2\sqrt{\beta }}$$

Dose response curves were also simulated using the mechanistic model for doses from 0 to 10 Gy for each particle type, and these dose response curves were used to calculate the model MID and *D*_10_ values. For X-ray exposures, these predictions were based solely on parameters obtained from mechanistic fitting in our previous work^24^, while for charged particle exposures a value of *E*_*DSB*_ was required. To obtain this, a nonlinear least squares regression was carried out, varying *E*_*DSB*_ to produce the best correlation between model predicted MID values and the experimentally observed proton MID values from the Paganetti dataset. Fits were carried out in python, using scipy’s optimize_curve routine. The PIDE dataset was not used within this fit, but was instead reserved to test the ability of the model to extrapolate between different systems, by calculating ion responses based on the *E*_*DSB*_ value obtained for protons without any fitting to the carbon data.

Finally, RBEs were calculated by comparing different ion exposures to their associated reference X-ray data. RBE is defined as the ratio of the dose required to produce a given endpoint by X-rays divided by the dose required to produce the same endpoint with ions. Thus, for example, the RBE at 10% survival for a given exposure can be defined as:

6 $$RBE=\frac{{D}_{10}^{X}}{{D}_{10}^{ion}}$$

General dose response curves were calculated for Fig. 7 in a similar fashion, with generic hamster (repair competent, G1 arrest deficient) and human (repair competent, G1 arrest competent) cell lines being simulated for a range of doses and different combinations of particle and LET. These resulting dose response curves were then fit to a standard LQ model curve to provide corresponding *α* and *β* parameters, which were compared to particle exposures after correction for X-ray sensitivity.

## Electronic supplementary material

Supplementary Information

Supplementary Dataset 1
