# Supplementary material for: A general mechanistic model enables predictions of the biological effectiveness of different qualities of radiation
Source: Sci Rep. 2017 Sep 7;7:10790. doi: 10.1038/s41598-017-10820-1 (PMC5589818; doi:10.1038/s41598-017-10820-1)
Supplement: Supplementary file 1 — Supplementary Information [file 41598_2017_10820_MOESM1_ESM.pdf]

A general mechanistic model enables predictions of the biological effectiveness of different qualities of radiation

Stephen J McMahon[1,2]*, Aimee L McNamara[2], Jan Schuemann[2], Harald Paganetti[2], Kevin M Prise[1]
1 Centre for Cancer Research and Cell Biology, Queen's University Belfast, Belfast, BT9 7AE, N. Ireland
2 Department of Radiation Oncology, Massachusetts General Hospital, 30 Fruit St, Boston, MA 02114, USA

Supplementary Information

RBE Model Comparison

To benchmark the model developed in this work against analytic RBE models, proton MID variations were calculated using three published models for each condition in the Paganetti RBE dataset. These models were those of Carabe et al, Wedenberg et al, and McNamara et al[1–3], which in each case define new α and β parameters for proton irradiation, based on the response of the cells to X-ray reference irradiations, the proton LET, and between 1 and 4 adjustable parameters.

In the Carabe model, quantities RBE_{max} and RBE_{min} are defined, corresponding to α_p/α_x and $\sqrt{\beta_p/\beta_x}$, respectively, as:

$$RBE_{max} = p_0 + \frac{p_1}{\left(\frac{\alpha}{\beta}\right)_x} LET_d = 0.843 + \frac{0.414}{\left(\frac{\alpha}{\beta}\right)_x} LET_d$$

$$RBE_{min} = p_2 + \frac{p_3}{\left(\frac{\alpha}{\beta}\right)_x} LET_d = 1.09 + \frac{0.0161}{\left(\frac{\alpha}{\beta}\right)_x} LET_d$$

Where LET_d is the dose-averaged Linear Energy Transfer, p_0, p_1, p_2 and p_3 were empirically fitted parameters, with values given as above. By contrast, the Wedenberg model assumes that β is constant, and that α scales linearly with LET_d, giving:

$$\frac{\alpha_p}{\alpha_x} = 1 + \frac{p_0 LET_d}{\left(\frac{\alpha}{\beta}\right)_x} = 1 + \frac{0.434\, LET_d}{\left(\frac{\alpha}{\beta}\right)_x}$$

$$\frac{\beta_p}{\beta_x} = 1$$

Where p_0 is again a model-specific fitting parameter. Finally, the McNamara model varies both α and β, similarly to the Carabe model, but assumes a different dependence on $\left(\frac{\alpha}{\beta}\right)_x$:

$$RBE_{max} = p_0 + \frac{p_1}{\left(\frac{\alpha}{\beta}\right)_x} LET_d = 0.99 + \frac{0.356}{\left(\frac{\alpha}{\beta}\right)_x} LET_d$$

$$RBE_{min} = p_2 + p_3 \sqrt{\left(\frac{\alpha}{\beta}\right)_x} LET_d = 1.1 - 0.0039 \sqrt{\left(\frac{\alpha}{\beta}\right)_x} LET_d$$

Using the published fitting parameters, these models were implemented in Python, and used to calculate α_p and β_p values for each experiment, which were in turn used to calculate the MID as described in the main text. RBE_{MID} was then calculated using these modelled proton parameters, and compared to that obtained from the experimentally observed proton responses. The resulting correlations are presented in Figure S1.

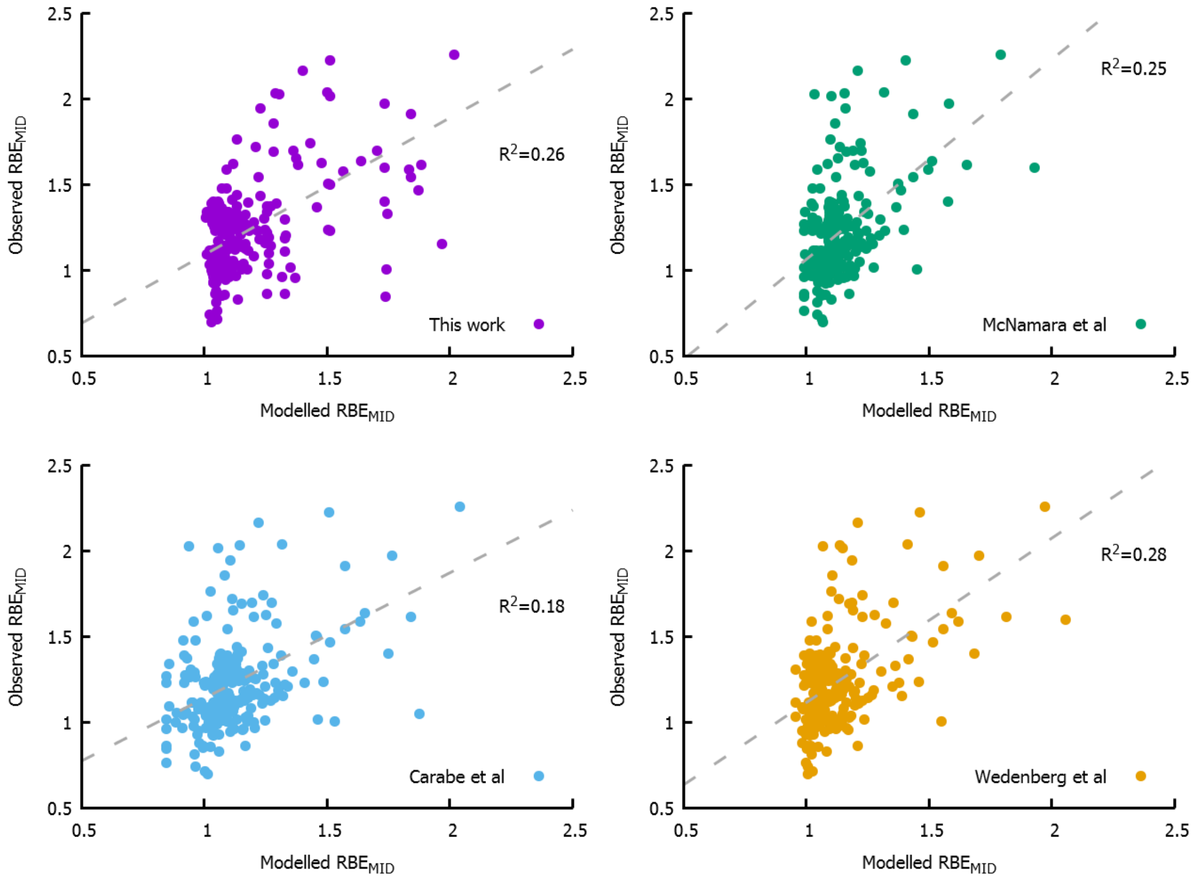

Supplementary Figure 1 Comparison of modelled RBE_{MID} values and experimental observations for a range of RBE models, including this work, McNamara et al, Carabe et al, and Wedenberg et al. It can be seen that, in all cases, the total correlation coefficient is less than 0.3, due to the high degree of experimental noise on these data points due to the small effect size. Due to this, it is challenging to demonstrate significantly superior predictive power considering proton data alone.

In all cases, while the different models do offer some predictive power and correlation with observed data, the degree of unexplained variance is very high, with no model having an R^2 of more than 0.3, even those which use measured cell-specific X-ray α and β values and multiple fitted empirical RBE parameters. This is comparable to the model presented in this work, which involves no cell-specific parameters and only a single RBE-related parameter.

Because RBE is defined as the ratio of two quantities whose uncertainty is large compared to their difference (for protons), random error is often significantly larger than the observed effect, which prevents any model from meaningfully fitting these data to high precision. As a result, other experiments with greater range (such as carbon ion RBE) must be used suitably test model predictions.

Carbon Ion Misrepair Data

Carbon ion radial energy depositions were processed in an identical fashion to those calculated for protons, and DSB distributions, interaction rates and overall misrepair rates are plotted in Figure S2.

It can be seen that the overall trends are very similar to those seen in proton exposures, with a rapid initial increase in DSB density with increasing radius, rapidly saturating out to equilibrium within a few hundred nm of the DSB. The DSB yields are significantly higher, however, reflecting the much higher LET of carbon ions than protons.

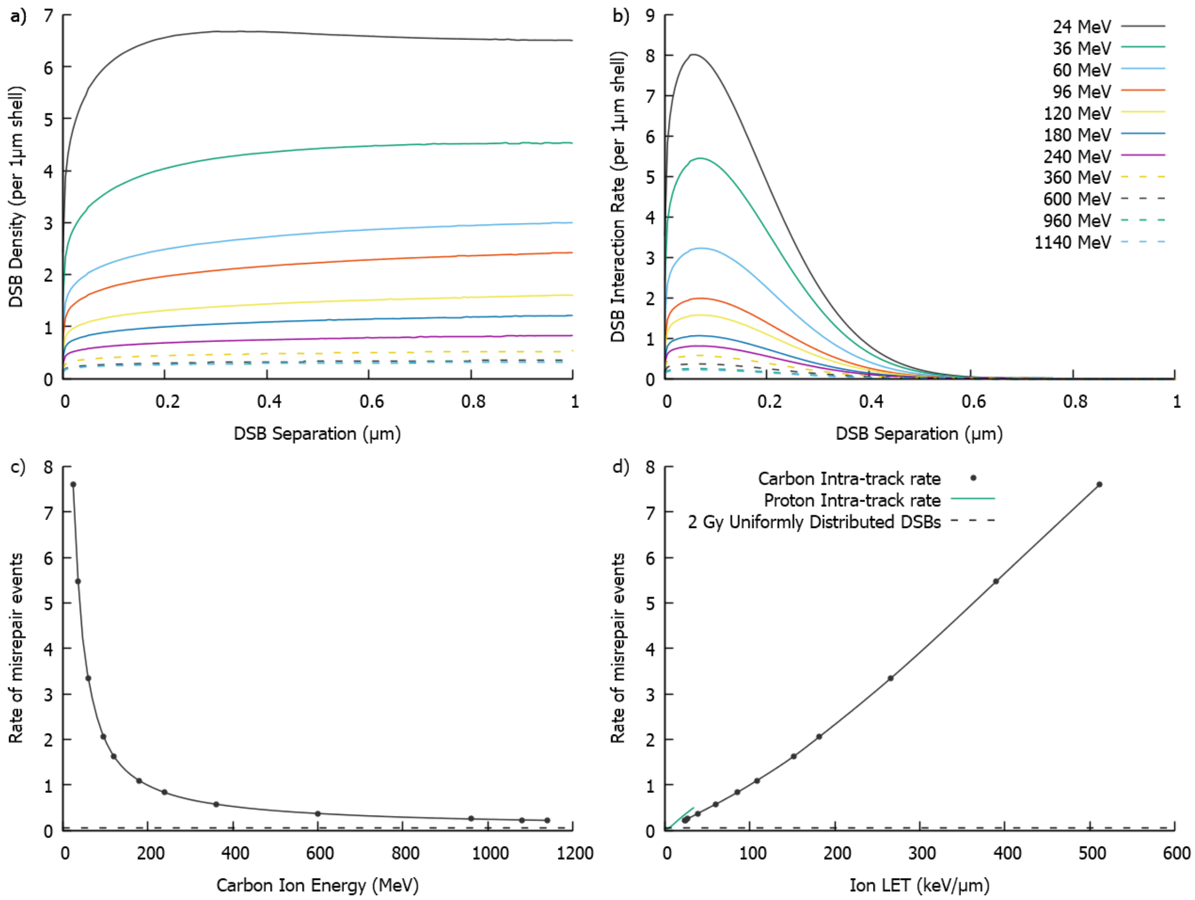

Supplementary Figure 2: Double Strand Break (DSB) distributions and resulting misrepair rates for carbon ions. As shown in the main text for protons, radial energy distributions can be used to calculate DSB distributions and misrepair rates. This is shown in a), with a rapid rise of DSB count with range as greater portions of the particle track are encompassed. Panel b) presents the resulting interaction rate, obtained by scaling the DSB density by $e^{-\frac{r^2}{2\sigma^2}}$*, showing most intra-track interactions still occur for DSBs separated by a few hundred nm. By integrating the interaction rates from b), the total interaction rate can be obtained for different particle energies. The rate is plotted as a function of either carbon ion energy (c) or LET (d). This rate increases steeply as the particle slows down, and is closely related to LET – although this relationship is slightly super-linear, due to the smaller total track radius at lower energies. The misrepair rate for 2 Gy of uniformly distributed DSBs is also plotted for comparison (dashed line), showing that for almost all LETs carbon ions drive significantly increased misrepair rates.*

As with protons, these high densities of DSBs also translate into increased intra-track misrepair rates, as shown in panels c and d. Misrepair rates once again increase significantly with increasing LET, again at a slightly super-linear rate, as the track width reduces as LET increases, further increasing overlap events. Notably, because of the higher mass and atomic number of carbon ions, the track width at any given energy is slightly greater for carbon ions than for proton ions, and thus at equal LET, the misrepair rate in carbon ion tracks is slightly lower than that for protons, illustrated by the proton misrepair curve plotted in Figure S2d.

References

1. Carabe, A., Moteabbed, M., Depauw, N., Schuemann, J. & Paganetti, H. Range uncertainty in proton therapy due to variable biological effectiveness. *Phys. Med. Biol* **57,** 1159–1172 (2012).

2. Wedenberg, M., Lind, B. K. & Hårdemark, B. A model for the relative biological effectiveness of protons: the tissue specific parameter α/β of photons is a predictor for the sensitivity to LET changes. *Acta Oncol. (Madr).* **52,** 580–8 (2013).

3. McNamara, A. L., Schuemann, J. & Paganetti, H. A phenomenological relative biological effectiveness (RBE) model for proton therapy based on all published *in vitro* cell survival data. *Phys. Med. Biol.* **60,** 8399–8416 (2015).
